# A review on Gene Ontology evaluations

**DOI:** 10.1093/database/baaf058

**Published:** 2025-09-24

**Authors:** Borja Pitarch, Mónica Chagoyen, Juan A G Ranea, Florencio Pazos

**Affiliations:** Computational Systems Biology, National Center for Biotechnology (CNB-CSIC), 28049 Madrid, Spain; Computational Systems Biology, National Center for Biotechnology (CNB-CSIC), 28049 Madrid, Spain; Department of Molecular Biology and Biochemistry, University of Málaga, 29071 Málaga, Spain; CIBER de Enfermedades Raras (CIBERER), Instituto de Salud Carlos III, 28029 Madrid, Spain; Institute of Biomedical Research in Malaga and Platform of Nanomedicine (IBIMA Platform BIONAND), 29071 Malaga, Spain; Spanish National Bioinformatics Institute (INB/ELIXIR-ES), 08034 Barcelona, Spain; Computational Systems Biology, National Center for Biotechnology (CNB-CSIC), 28049 Madrid, Spain

## Abstract

The analysis of the large and heterogeneous datasets that characterize modern biology demands systems capable of representing biological knowledge in a formal, standardized manner. The most widely used structured vocabulary in molecular biology and biomedicine is the Gene Ontology (GO). Although employed in many different data analysis tasks, its main application is in the functional annotation of gene products and the subsequent functional enrichment analysis of large gene/protein datasets derived from omics experiments. This key position in modern biology means the GO is subject to continuous scrutiny and improvement, not only by its developers but by the community in general. GO’s vocabulary, structure, and gene annotations have been evaluated from multiple points of view, revealing important insights into how we organize our biological knowledge, the associated problems, and strategies for overcoming them. These evaluations range from technical analyses of its ontological architecture to assessments of its effectiveness in supporting biological research. This process ensures that this essential resource is updated and adapted to the new challenges posed by modern biology. In this review, we summarize some key evaluations that the GO has undergone over time.

## Introduction—the Gene Ontology

**Figure 1.**
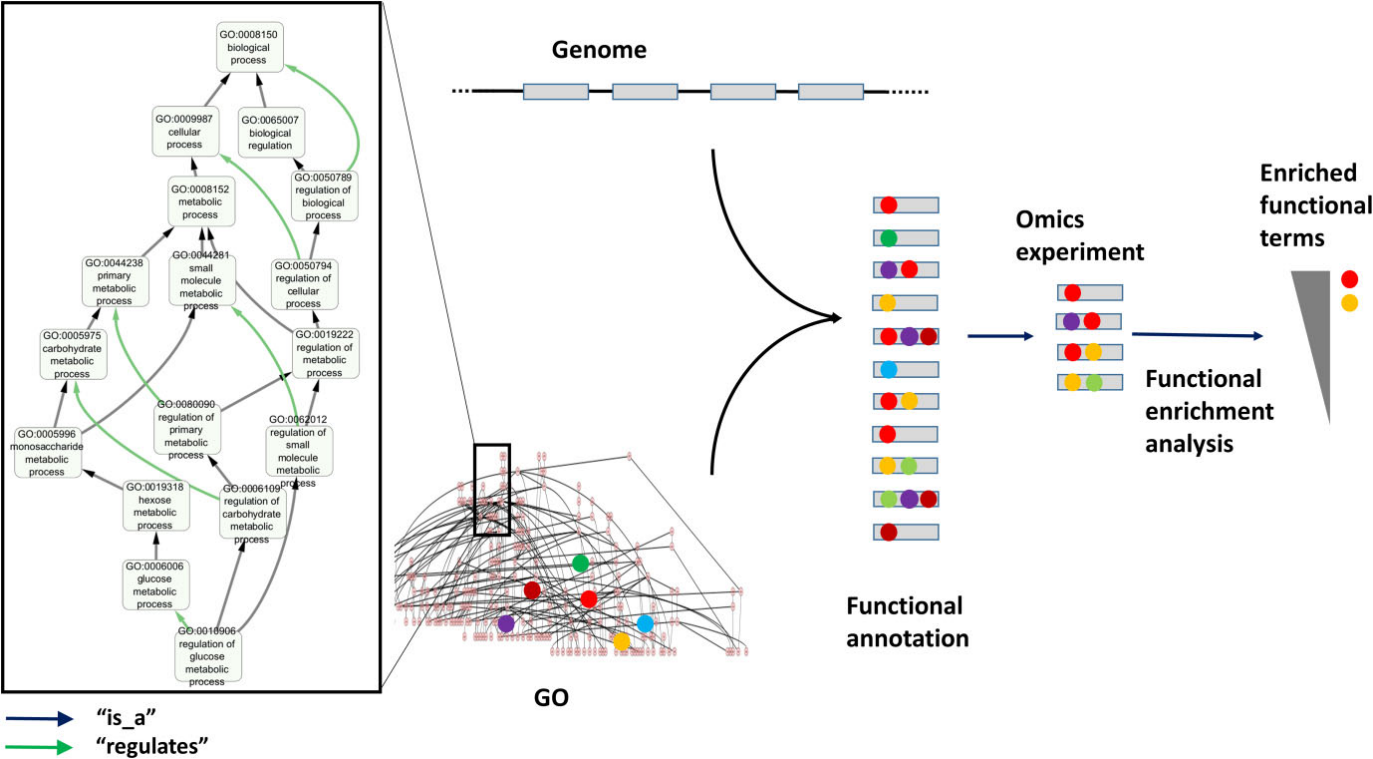
GO structure and usage for functional annotation and enrichment analysis. The left panel displays a subgraph of the GO ‘biological process’ DAG, with the term ‘regulation of glucose metabolic process’ (GO: 0010906) and all its ancestors up to the root of the subontology (‘biological process’, GO:1118150). The ‘is_a’ and ‘regulates’ relationships connecting these terms are indicated. Functional annotation of a set of genes (e.g. a newly sequenced genome) involves assigning GO terms to genes (coloured balls) based on different evidences. Once annotated, gene sets (e.g. those differentially expressed in an omics experiment) can be functionally analyzed by identifying GO terms statistically overrepresented in the set relative to a background (e.g. all genes of the genome).

In the context of this review, an ‘ontology’ can be defined as a formal way of representing knowledge so that it can be handled by computers [1]. In the simplest form, an ontology consists of entities (terms of a controlled vocabulary), linked by relationships of different nature [2] (Fig. 1). Given the complexity of biological knowledge, ontologies are becoming increasingly popular in this area [3]. There are many ontologies in the biomedical domain, designed to represent different types of biological and molecular knowledge [4,5]. At the time of writing, the BioPortal repository of biomedical ontologies [6] (https://www.bioontology.org/) lists over 1150 ontologies. One of these, the Gene Ontology (GO), was originally developed to represent knowledge about the functions of gene products at the cellular level, although it is now being extended to encompass other biological levels, including organisms [7].

To understand the characteristics and potential limitations of GO, it is helpful to begin with the problem of gene/protein functional annotations. Protein function is a complex and multidimensional concept [8]. While protein sequences and structures are univocal concepts, easy to define, represent, and quantify (e.g. sequence or structural similarity between two proteins), and stable in time, that is not true for function. In contrast to sequence and structure, there is not univocal definition or representation of protein function grounded into molecular principles. Our definitions of ‘protein function’ are tied to the molecular biology toolbox and evolve over time with shifting conceptual perspectives on molecular phenomena. As a result, multiple vocabularies for describing different aspects of protein function have historically coexisted [9]. Most major databases developed their own functional vocabulary, typically as ‘flat’ unstructured lists of terms, meaning that they defined sets of functional terms (vocabulary) without specifying relationships or hierarchies among them. In this context, the Gene Ontology Consortium established GO around 1998 (formally published in 2000) [10], with the goal of creating a structured vocabulary for representing the diverse functional aspects of (originally) eukaryotic genes.

From its inception, GO was divided in three subontologies that describe (in principle, orthogonal) aspects of the protein/gene function: ‘molecular function’ (GO:MF), which includes terms describing activities carried out by a gene products at the molecular level (e.g. ‘chaperone’, ‘GTPase’, and ‘alcohol dehydrogenase’); ‘biological process’ (GO:BP), which relates to the broader biological objectives a protein contributes to (e.g. ‘signalling’ and ‘metabolic process’); and ‘cellular component’ (GO:CC), which defines the subcellular locations where a protein performs its function (e.g. ‘cytoplasm’ and ‘nucleus’). These three aspects are, in principle, orthogonal. For example a GTPase (GO:MF) may function in either a metabolic pathway or a signalling cascade (GO:BP), and may do so in in distinct cellular compartments (GO:CC). Within each subontology, terms are linked by various types of relationships (e.g. ‘is_a’, ‘part_of’, ‘regulates’). These relationships are directed (e.g. A *part_of* B is not equivalent to B *part_of* A, and they form a directed graph without cycles [formally a ‘directed acyclic graph’ (DAG)] (Fig. 1). The hierarchical nature of these relationships allows navigating from general functional aspects (e.g. ‘metabolic process’) down to more specific ones (e.g. ‘4-nitrophenol metabolic process’) (Fig. 1).

The terms and their relationships within GO are defined by the GO Consortium, based on expert knowledge and community feedback. Once the ontology is established, a gene product (protein) can be functionally annotated by assigning one or more GO terms from the three subontologies (Fig. 1). Such annotations may be supported by various types of evidence, ranging from direct experimental data to automatic inference based on sequence similarity.

From its initial release, GO rapidly gained popularity and soon became the *de facto* standard for protein functional annotation. With the advent of omics technologies, one of the most widely used applications of protein functional annotations, and GO annotations in particular, is the analysis of large collections of proteins via ‘functional enrichment analysis’ [11]. These analyses identify functional terms (e.g. GO terms) that are overrepresented in a list of genes or proteins (e.g. those overexpressed in an experiment) relative to a background (e.g. all genes in the genome of interest). As a result, long gene lists can be ‘distilled’ into a smaller set of biologically meaningful terms corresponding, for example, to pathways (GO: BP terms) whose genes tend to be overexpressed in the experiment (Fig. 1).

GO’s widespread used has fuelled its expansion, and today it contains over 40 000 terms, used to annotate 1.5 million gene products across more than 5000 species [7]. It plays a central role in omics data analysis, and it is used daily by thousands of researchers. GO has been cited more than 159 000 times and used in high-impact studies such as the ENCODE project [12] or the COVID-19 host-genetic initiative [13]. As a result, it remains under continuous scrutiny by both its developers and the broader research community. This review aims to provide an overview of the main types of evaluations to which GO has been subjected, the insights gained, and the changes or alternatives proposed to address its limitations. We focus specifically on the evaluations of the GO itself, rather than on GO-based functional annotations, although the two are difficult to separate in some evaluations. Our goal is not to comprehensively catalogue all existing evaluations, but to highlight representative examples of each major approach. Similarly, we do not aim to review all aspects of GO, but rather to concentrate on its evaluation and assessment. Topics such as GO’s structure, its use in gene functional annotation, and its development history are only briefly discussed to provide context for understanding the evaluations.

### Self-revisions and domain-specific adaptations

The GO Consortium makes a great effort to evaluate the limitations and missing components of the resource in order to update and adapt it to our evolving understanding of biology and the needs of the community. Although this is by no means an exhaustive list, some key milestones in this ongoing process are outlined below.

In the early stages, developers recognized that the first obvious steps following GO’s inception were to expand the ontology with new terms, based on community input [14], and to increase the number of functional annotations to ensure coverage of major model organisms [15]. Needless to say, the expansion in terms and annotations is ongoing. After this initial phase of expansion, greater emphasis was placed on improving the structure of the ontology itself: new types of relationships between terms were introduced, and relationships between terms of different subontologies were allowed [16]. In 2015, terms describing multiorganism processes (e.g. host–parasite interactions) were added [17]. Two years later, direct functional annotations of microRNA genes were incorporated [18]; previously, these had been annotated indirectly by transferring the annotations of their target protein-coding genes. More recently, efforts have focused on reevaluating older annotations, and ‘casual activity models’ (GO-CAMs) were introduced [19]. GO-CAMs go beyond standard GO annotations by integrating them into structured models of biological systems, which can, for example, be used in network-modelling approaches [20].

As GO became more widely used by researches from diverse fields, it became apparent that for certain biological systems it lacked the necessary level of detail and granularity. The GO Consortium emerged from a collaboration among three databases focused on model eukaryotic organisms (*Drosophila*, mouse, and yeast) [10], and thus initially reflected the functions and biological processes described in those organisms at the cellular level. Although GO was later extended to include other model organisms, it still lacked detailed coverage for specific biological systems of interest to some communities, prompting targeted expansions in those areas. For example, in 2011 the number of terms related to heart development were expanded from 12 to 280 [21], along with the corresponding relationships and gene annotations. Three years later, a major effort to incorporate knowledge on kidney development led to the addition of 522 new terms and 940 protein functional annotations [22]. Similar expansions—involving new terms, annotations, or both were done for immunology [23], muscle biology [24], blood–brain barrier [25], and Parkinson’s disease [26], among others. These expansions have enabled, among other benefits, the application of GO-based functional enrichment analysis to omics datasets in these systems.

Annotation expansions are usually accompanied by studies assessing their impact on GO’s primary application: functional enrichment analysis (see ‘biological evaluations’ below). A recent example is the GO Consortium’s evaluation of the effects of expanding human gene annotations on enrichment analysis [27].

## Ontology evaluation

A few years after the creation of the GO, Smith *et al*. [28] assessed its early achievements and challenges, emphasizing the need to improve consistency to enhance its applicability in computational biology. According to these authors, the GO Consortium prioritized its practical usability (aimed to the functional annotation of gene products in model organism databases) over ontological rigour or logical formalization. This pragmatic approach facilitated the rapid expansion of biological concepts but led to inconsistencies and ambiguities in both GO terms and their relations. For example, defining molecular functions as activities rather than as functions (i.e. the potential for activity) introduced conceptual confusion. One notable case was the term ‘structural molecule’, which referred to a molecule rather than an activity, contradicting the ontology’s framework. The GO Consortium partially addressed this by explicitly adding the concept of ‘activity’ in most molecular function terms, with the exception of binding terms.

From a practical perspective, Yeh *et al*. [29] evaluated the suitability of Protégé (https://protege.stanford.edu/), an open-source ontology editor, to maintain and develop GO. They underscored its potential for detecting redundant hierarchical annotations, tracking ontology changes, and capturing implicit information (such as ‘sensu’ terms, which specify concepts within specific taxonomic contexts). They also proposed adding new relations across the three GO subontologies, such as ‘part_of_process’ (linking molecular functions and biological processes) and ‘occurs_at_component’ (linking biological processes and cellular components). At that time, intersubontology relationships had not yet been implemented in GO.

Ogren *et al*. [30] analysed GO term names and revealed frequent substring relationships—where one term is included within another)—as well as recurring words and syntactic patterns. Some of these patterns (e.g. ‘regulation’) suggested implicit relationships between terms that were not explicitly represented in the GO structure at the time.

As researchers began applying GO for other purposes beyond functional annotation, new challenges surfaced. Smith *et al*. [31] identified several limitations when using GO for tasks such as automated reasoning, semantic data integration, and text mining. Chief among these were the absence of inter-subontology relations and problems with various *de facto* definitions for ‘part_of’ and ‘is_a’ relations (the only two classes of relationships at that time). The authors recommended introducing a standardized set of well-defined relation types. This issue was later addressed with the creation of the OBO Relation Ontology [32], a curated set of relation types for consistent use across OBO Foundry ontologies. The GO Consortium subsequently adopted this relation ontology to define relationships within GO.

Most recent work on the evaluating GO’s relational structure includes: the identification of redundant and missing links by analysing the compositional structure of GO terms [33]; detection of redundant hierarchical relations across the GO and other biomedical ontologies [34]; lexical approaches to identifying subtype (‘is_a’) inconsistencies [35]; evidence-based lexical pattern methods for predicting potentially missing relations [36]; machine learning approaches to infer missing ‘is_a’ relationships [37]; and inference of novel relationships between terms based on network topology [38].

While most of studies have focused on relationships between terms, fewer efforts have addressed the intrinsic quality of terms themselves. Kholer *et al*. [39] proposed two quantitative metrics to assess this: *circularity*, measuring the extent to which a term appears in its own definition; and *intelligibility*, measuring how clearly and accurately terms and definitions can be understood by users. Verspoor *et al*. [40] developed a method to identify univocally violations, i.e. cases where similar concepts are expressed using inconsistent linguistic conventions. More recently, Mayor and Robinson critiqued GO’s status as ‘controlled vocabulary’, noting its departure from established library and information science (LIS) standards, such as ISO2788 and NISO Z39.19i [41]. Compared to LIS-compliant vocabularies, GO showed several deficiencies: weak synonym control, lack of documented warrant (justification) for term selection, and the creation of nonstandard terms and definitions. Its structure favoured ontological realism (where terms correspond to an objectively real biological entities or processes) over exhaustive synonym coverage. The absence of scope notes (explanatory notes that define terms or provide guidance on their use) further hindered precise interpretation of terms. Finally, Ochs *et al*. [42] used two types of abstraction networks (area taxonomy and partial-area taxonomy) to identify groups of anomalous terms in the GO biological process subontology. By analysing root-area terms deep within the ontology, terms lacking lateral relationships, those modelled with diverse relation types and overlapping terms, they were able to find several kind of errors like incorrect logical modelling, missing or incorrect parents, and missing relationships.

## Biological evaluations

As the main practical use of GO is as a functional annotation resource for performing enrichment analysis on large gene or protein sets, most biological evaluations aim to assess its performance in that task. This typically involves evaluating the enrichment results using ‘gold standard’ datasets—cases in which the annotations expected to be enriched (e.g. affected biological pathways) are known or hypothesized based on the underlying experiment. A general limitation of these approaches is that they often do not disentangle the influence of the ontology itself (i.e. its terms and relationships) from that of the annotations on the enrichment results.

One of the first works in this area was conducted by Groß *et al*. [43]. Using both simulated and experimental data, they evaluated how changes in the GO’s structure and annotations influenced enrichment analysis results. The authors concluded that the results were surprisingly stable when semantic similarity between terms was considered (e.g. when a term was not directly recovered in the analysis but a semantically ‘similar’ one appeared instead). Their analysis also revealed that structural changes in GO were not uniformly distributed: some parts of the ontology evolved more extensively than others over time. Clarke *et al*. [44] assessed the performance of GO (ontology and annotations) in enrichment analysis tasks at different historical time points, by applying enrichment analysis with various archived versions of GO and their corresponding annotations. The main conclusion was that the performance improved over time. However, this study evaluated only specific ontology regions relevant to their benchmark datasets, selected based on expert knowledge. More recently, a similar study examined enrichment results derived from a large gene expression dataset related to 104 diseases, using GO versions spanning one decade [45]. In contrast to the earlier work, this study found that enrichment results were highly dependent on the specific GO version used and showed low consistency across different versions. The GOtrack server [46] allows users to observe how enrichment results for a list of genes or proteins of interest change over time across different GO versions and their associated functional annotations, for nine model organisms. Based on 2500 human gene lists, the authors reported that enrichment results remained stable over time for half of these lists.

The GO Consortium itself has investigated the impact of large annotation expansions on functional enrichment analysis, as illustrated in a recent study on human gene annotations [27].

Initiatives such as the CAFA challenge (Critical Assessment for Functional Annotation) [47], which benchmark protein function prediction methods, can also inform GO evaluations. Although these efforts focus on assessing predictive algorithms that assign GO terms to proteins, their outcomes provide indirect insights into how well GO captures the functional landscape.

Another approach to assessing GO’s biological coherence involves examining how well its structure and annotations align with experimental datasets that reveal biologically related groups of genes or proteins. For the ‘biological process’ subontology (GO:BP), it is expected that proteins annotated with the same GO:BP term will cluster in biological networks representing functional associations, such as the interactome [48] or coexpression networks [49]. This strategy enables the quantification of the biological coherence of individual GO:BP terms as well as that of term pairs related within the ontology (i.e. siblings or parent–child relationships). The main finding was that although most terms and relationships were ‘biologically coherent’ (i.e. they reflected the experimental relationships observed in the interactome) some were not [50]. This approach also allows the identification of term pairs that are not related within the ontology but should be functionally ‘close’ based on experimental data, as their annotated proteins tend to cluster in the interactome, as well as pairs of terms that in spite of being close in the ontology, represent biologically distant concepts.

## Proposed changes in the ontology and alternative ontologies

**Figure 2.**
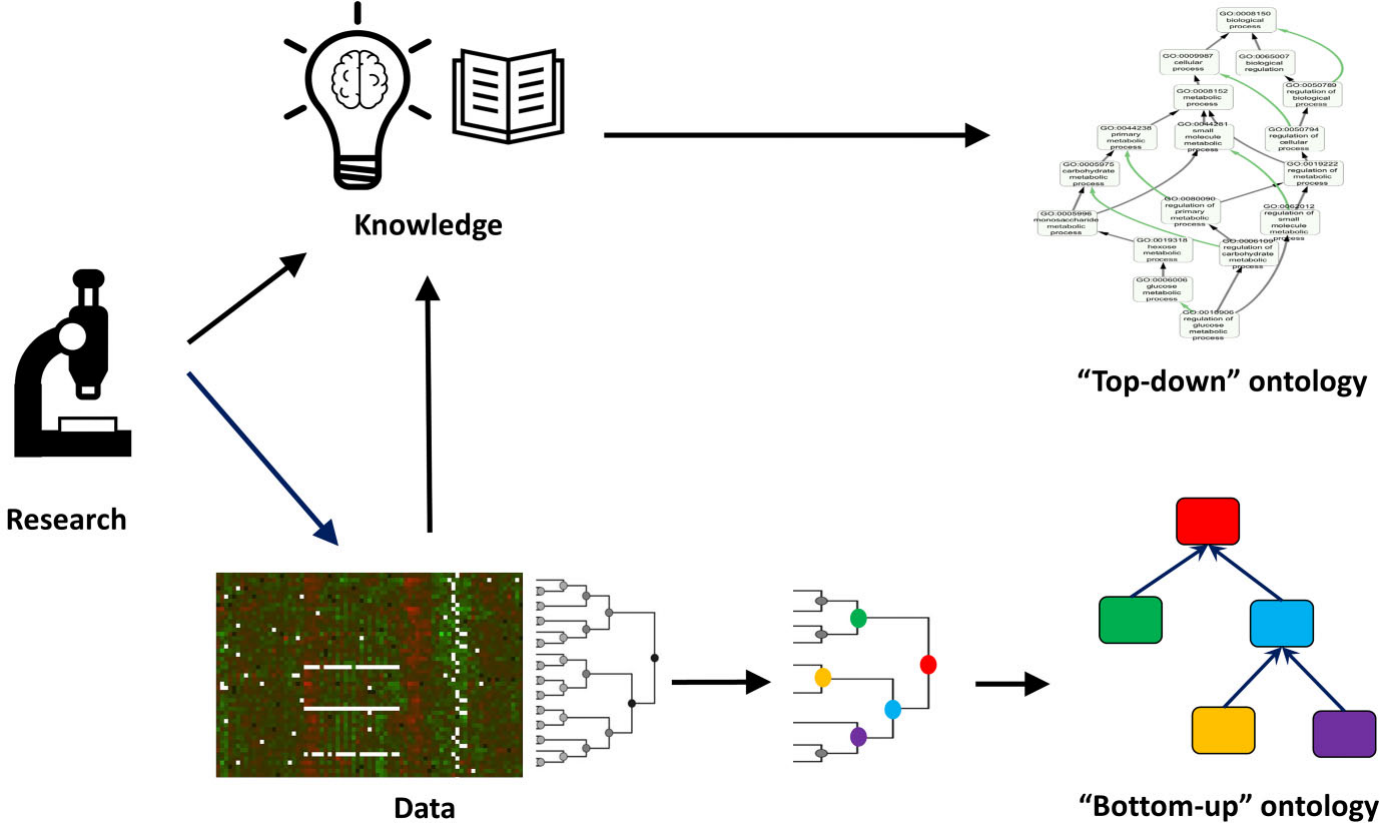
‘Top-down’ versus ‘bottom-up’ ontology construction. ‘Top-down’ ontologies are created based on expert-curated knowledge. In contrast, ‘bottom-up’ ontologies are derived from data, based on the inherent structure or clustering patterns observed. For example, an ontology can be constructed from clusters of genes identified from expression or protein interaction data.

The results of some of the studies discussed above have led researchers to propose alternative ‘ontologies’ (that is, sets of biological terms connected by some form of relationship) to complement the GO. For instance, the partial inconsistencies observed between GO and interactome data [50] suggest the potential value of developing ‘bottom-up’ ontologies derived directly from experimental data. The central idea is that unsupervised clustering of such data could naturally define ontology terms and their relationships (for example, through topological clusters of protein–protein interaction networks or coexpression modules) (Fig. 2).

A notable example is the NeXO ontology, developed by Ideker and collaborators, in which both terms and their relationships are inferred from the structure of the interactome [51,52]. While many NeXO terms can be mapped to existing GO terms, others represent novel concepts supported by independent lines of evidence, such as genetic interaction profiling and chemogenomic experiments.

An intermediate approach is to generate new terms from experimental data and integrate them into to the existing GO structure, rather than creating an entirely separate ontology. This strategy is exemplified by GOExtender [53], a method that identifies candidate GO terms based on experimental network data, connects them to the existing GO DAG, and simultaneously annotates genes with these newly proposed terms. A more sophisticated solution that use machine learning and experimental data to extend GO is implemented in Unicorn [54]. Unicorn is trained on existing segments of GO and the associated gene experimental data to predict novel expansions of the ontology. Its primary advantage over earlier approaches lies in its ability to extend all three GO subontologies, including ‘molecular function’ (GO:MF), whereas methods like NeXO and GOExtender are mostly limited to ‘cellular component’ (GO:CC) and/or ‘biological process’ (GO:BP), where the relationship between terms and interactome clusters is more apparent.

Apart from these ‘data-driven’ ontologies, another line of ontology expansion involves the automatic mining of scientific literature [55]. For example, Chagoyen *et al*. [56] explored the identification of relationships between GO biological processes by analysing textual similarities among the PubMed abstracts linked to each term. This strategy not only, validates explicit and implicit connections already present in GO but also reveals potential new relationships among GO: BP terms that are not currently captured in the ontology.

## Discussion

Given the complexity of biological data and knowledge, ontologies have become increasingly popular in molecular biology and biomedicine. They provide a standardized, controlled, and structured vocabulary to represent biological knowledge, which allows interoperability, data integration, and, in general, facilitates the computational handling of biological data. The evaluation of ontologies is a fundamental component of their development/maintenance cycle [57]. In a recent review, Amith *et al*. [58] found that from a sample of 200 biomedical ontologies they analysed, only 15 reported an evaluation procedure in their original publications.

**Figure 3.**
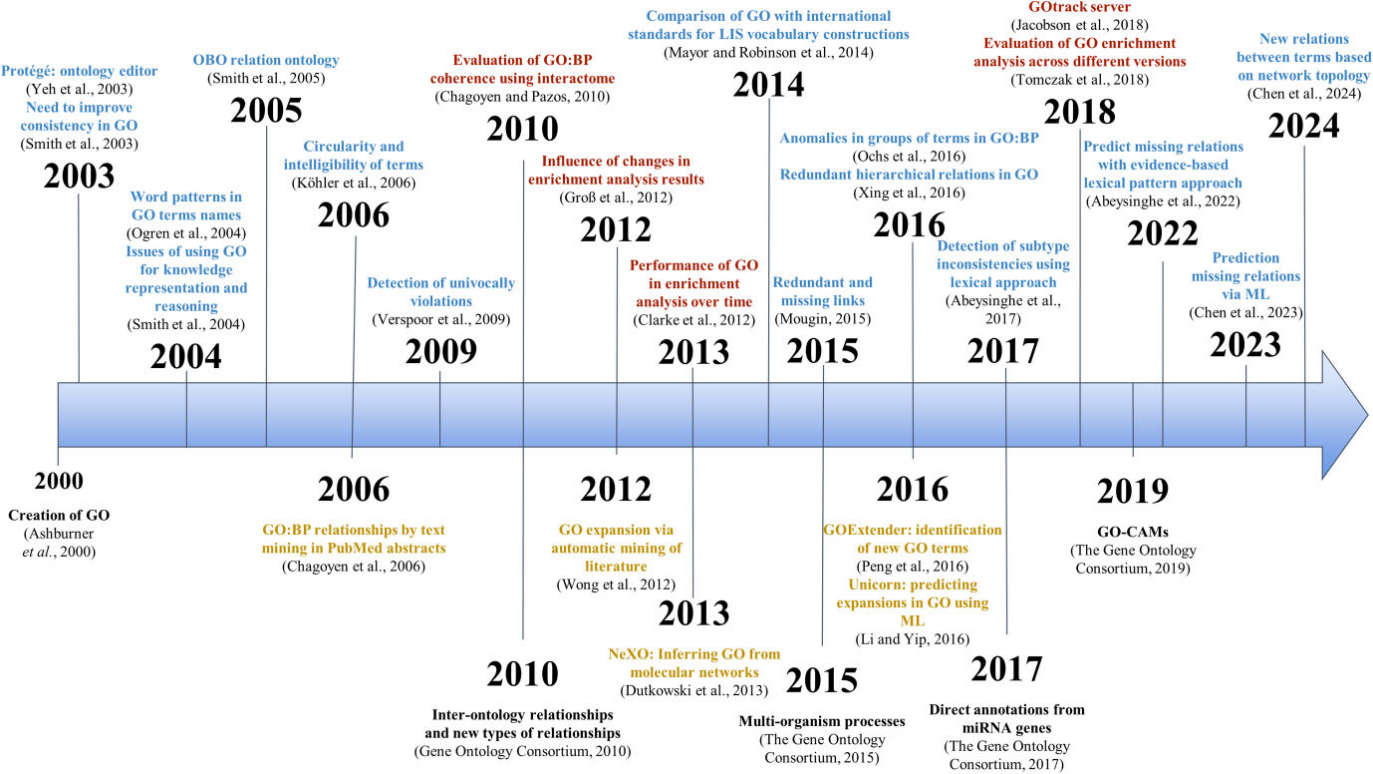
Timeline of key GO milestones and evaluation efforts. The upper section of the timeline shows selected evaluations studies discussed in the text (red: biological evaluations; blue: ontological evaluations). The lower section shows major milestones in the development of GO (black), along with proposed modifications and alternative ontology initiatives (yellow).

GO aims to encode our biological knowledge and, consequently, it is dynamic and changes with time. It has been continuously updated and expanded since its inception, in collaboration with the community. See Fig. 3 for an overview of the main milestones, changes, and evaluations discussed in this review.

From its inception, the GO prioritized its practical usability—primarily for gene annotation and functional enrichment analysis—over formal ontological aspects. That focus kept it simple enough to be used without a deep understanding of its structure or even its original goal [59]. This utilitarian view has been beneficial and undoubtedly contributed to GO’s widespread adoption, but it also led to problems that emerged in specific contexts. Indeed, many problems with GO are a consequence of using the resource for purposes beyond its original goal, rather than inherent flaws in the resource itself [50,60]. GO’s ontological complexity can also be confusing for users unfamiliar with its structure—for example when numerous seemingly redundant terms (e.g. parent/child terms) appear in an enrichment analysis, requiring the development of *Ad hoc* tools to simplify and summarize results (e.g. [61]).

A consequence of this prioritization of practical usability for gene functional annotation was that the development of more technical ontological aspects was delayed, and most efforts were devoted to improving the annotation-related features. This is also reflected in the evaluations carried out: most ontological evaluations are relatively old, and it appears that the community now places less emphasis on ontological aspects, focusing instead on the biological evaluations (Fig. 3). Nevertheless, ontological aspects should not be overlooked, as these are fundamental to using GO as a knowledge representation framework and ultimately influences gene annotations as well.

The GO structure is highly heterogeneous, with some parts of the DAG—those representing better-understood biological domains—being more densely populated with terms and connections than others. As a result, the same network motif in the DAG (e.g. a parent term linked to two siblings) can represent different degrees of functional similarity depending on the specific region of the DAG.

In line with its main application, most of the GO evaluations focused on the gene product annotations [e.g. coverage, reliability, evidences, and temporal evolution (e.g. [59,62])], rather than on the ontology’s structure itself. These annotation-focused aspects are not explicitly covered in this review, although annotations also play a role in the ontology evaluations discussed here, particularly in the biological evaluations. Most of the biological evaluations assess the effect of changes in the ontology (both structure and annotations) on the results of functional enrichment analyses. Significant impacts on enrichment analysis are concerning, as they may affect the interpretation and reproducibility of findings—especially when comparing analyses performed using different versions of GO. In general, older evaluations tend to be more optimistic about the stability of enrichment results across GO versions compared with more recent studies.

Regarding protein functional annotations, GO is a ‘top-down’ schema, where functional terms and their relationships are defined by expert knowledge. As such, it includes terms that reflect groups of genes linked by underlying molecular or evolutionary mechanisms, as well as others that are categorizations lacking this molecular basis. These two types of groups in classification schemas in general have been termed ‘classes’ and ‘categories’, respectively [63]. Categories are useful for organizing knowledge and do not pose a problem, as long as they are recognized as such and not used for the same purposes as classes (e.g. in enrichment analysis). Agreement with clusters of gene relationships obtained from experimental data can help distinguish classes from categories [50], and may eventually lead to the development of ‘bottom-up’ ontologies purely based on data, agnostic to the available knowledge [52] (Fig. 2). These data-driven ontologies complement expert-defined schemas and offer the advantage of being automatically derived from data, while the latter are manually curated and therefore cannot keep pace with the rapid accumulation of biological knowledge and data.

On the contrary, in data-driven ontologies, the terms and relationships may lack informative ‘labels’, complicating the interpretation of results as those from enrichment analysis. Consequently, we do not expect these ontologies to gain widespread practical use, as users value GO’s informative term names, even if some lack a clear molecular rationale. The use of text-mining approaches to automatically derive ontologies from the scientific literature may help address this limitation. Deep learning methods are already being used to extract knowledge from text and to construct ontologies from it (e.g. [64]). Indeed, the ongoing revolution in deep learning will undoubtedly enhance GO in many ways and will also contribute to its evaluation.

Altogether, these evaluations demonstrate that GO is a tremendously useful resource, albeit with certain limitations. Its central role in biology makes that it is continuously scrutinized and evaluated from multiple perspectives. Many of the shortcoming identified in past evaluations have subsequently been addressed by the GO Consortium. As GO has grown in popularity, it has become the de facto standard for representing protein function. The fact that it is almost the only alternative should not preclude its continuous scrutiny and assessment, but the other way around. Another important issue yet to be addressed is the need to make the results of GO evaluations available to end users through web servers or databases, which could be linked directly to GO. In this way, users could access, for example, confidence estimates associated with GO-based outputs, including terms and relationships.
